# Supplementary material for: A Critical View of the Application of the APEX Software (Aqueous Photochemistry of Environmentally-Occurring Xenobiotics) to Predict Photoreaction Kinetics in Surface Freshwaters
Source: Molecules. 2019 Dec 18;25(1):9. doi: 10.3390/molecules25010009 (PMC7017383; doi:10.3390/molecules25010009)
Supplement: Supplementary file 1 [file molecules-25-00009-s001.zip › 3.2.4_gcc-4.4.0.zip!/3.2.4_gcc-4.4.0/3.2.4_gcc-4.4.0/doc/octave/html/interpreter/Manipulating-Strings.html]

Manipulating Strings - Untitled


Next: String Conversions,
Previous: Comparing Strings,
Up: Strings


---

### 5.5 Manipulating Strings

Octave supports a wide range of functions for manipulating strings.
Since a string is just a matrix, simple manipulations can be accomplished
using standard operators. The following example shows how to replace
all blank characters with underscores.

```
     quote = ...
       "First things first, but not necessarily in that order";
     quote( quote == " " ) = "_"
     => quote =
         First_things_first,_but_not_necessarily_in_that_order
```

For more complex manipulations, such as searching, replacing, and
general regular expressions, the following functions come with Octave.

— Function File: **deblank** (s)  
> Remove trailing blanks and nulls from s. If s
> is a matrix, deblank trims each row to the length of longest
> string. If s is a cell array, operate recursively on each
> element of the cell array.

— Function File: **strtrim** (s)  
> Remove leading and trailing blanks and nulls from s. If
> s is a matrix, strtrim trims each row to the length of
> longest string. If s is a cell array, operate recursively on
> each element of the cell array. For example:
>
> ```
>           strtrim ("    abc  ")
>                => "abc"
>           
>           strtrim ([" abc   "; "   def   "])
>                => ["abc  "; "  def"]
> ```

— Function File: **strtrunc** (s, n)  
> Truncate the character string s to length n. If s
> is a char matrix, then the number of columns is adjusted.
>
> If s is a cell array of strings, then the operation is performed
> on its members and the new cell array is returned.

— Function File: **findstr** (s, t, overlap)  
> Return the vector of all positions in the longer of the two strings
> s and t where an occurrence of the shorter of the two starts.
> If the optional argument overlap is nonzero, the returned vector
> can include overlapping positions (this is the default). For example,
>
> ```
>           findstr ("ababab", "a")
>                => [1, 3, 5]
>           findstr ("abababa", "aba", 0)
>                => [1, 5]
> ```
>
>
>
>
> **See also:** strfind, strmatch, strcmp, strncmp, strcmpi, strncmpi, find.

— Function File: idx = **strchr** (str, chars)  
— Function File: idx = **strchr** (str, chars, n)  
— Function File: idx = **strchr** (str, chars, n, direction)  
> Search for the string str for occurrences of characters from the set chars.
> The return value, as well as the n and direction arguments behave
> identically as in `find`.
>
> This will be faster than using regexp in most cases.
>
>
>
> **See also:** find.

— Function File: **index** (s, t)  
— Function File: **index** (s, t, direction)  
> Return the position of the first occurrence of the string t in the
> string s, or 0 if no occurrence is found. For example,
>
> ```
>           index ("Teststring", "t")
>                => 4
> ```
>
> If direction is "first", return the first element found.
> If direction is "last", return the last element found.
> The `rindex` function is equivalent to `index` with
> direction set to "last".
>
> **Caution:** This function does not work for arrays of
> character strings.
>
>
>
> **See also:** find, rindex.

— Function File: **rindex** (s, t)  
> Return the position of the last occurrence of the character string
> t in the character string s, or 0 if no occurrence is
> found. For example,
>
> ```
>           rindex ("Teststring", "t")
>                => 6
> ```
>
> **Caution:** This function does not work for arrays of
> character strings.
>
>
>
> **See also:** find, index.

— Function File: idx = **strfind** (str, pattern)  
— Function File: idx = **strfind** (cellstr, pattern)  
> Search for pattern in the string str and return the
> starting index of every such occurrence in the vector idx.
> If there is no such occurrence, or if pattern is longer
> than str, then idx is the empty array `[]`.
>
> If the cell array of strings cellstr is specified instead of the
> string str, then idx is a cell array of vectors, as specified
> above. Examples:
>
> ```
>           strfind ("abababa", "aba")
>                => [1, 3, 5]
>           
>           strfind ({"abababa", "bebebe", "ab"}, "aba")
>                => ans =
>                   {
>                     [1,1] =
>           
>                        1   3   5
>           
>                     [1,2] = [](1x0)
>                     [1,3] = [](1x0)
>                   }
> ```
>
>
>
>
> **See also:** findstr, strmatch, strcmp, strncmp, strcmpi, strncmpi, find.

— Function File: **strmatch** (s, a, "exact")  
> Return indices of entries of a that match the string s.
> The second argument a may be a string matrix or a cell array of
> strings. If the third argument `"exact"` is not given, then
> s only needs to match a up to the length of s. Nul
> characters match blanks. Results are returned as a column vector.
> For example:
>
> ```
>           strmatch ("apple", "apple juice")
>                => 1
>           
>           strmatch ("apple", ["apple pie"; "apple juice"; "an apple"])
>                => [1; 2]
>           
>           strmatch ("apple", {"apple pie"; "apple juice"; "tomato"})
>                => [1; 2]
> ```
>
>
>
>
> **See also:** strfind, findstr, strcmp, strncmp, strcmpi, strncmpi, find.

— Function File: [tok, rem] = **strtok** (str, delim)  
> Find all characters up to but not including the first character which
> is in the string delim. If rem is requested, it contains the
> remainder of the string, starting at the first delimiter. Leading
> delimiters are ignored. If delim is not specified, space is
> assumed. For example:
>
> ```
>           strtok ("this is the life")
>                => "this"
>           
>           [tok, rem] = strtok ("14*27+31", "+-*/")
>                =>
>                   tok = 14
>                   rem = *27+31
> ```
>
>
>
>
> **See also:** index, strsplit.

— Function File: [s] = **strsplit** (p, sep, strip\_empty)  
> Split a single string using one or more delimiters and return a cell
> array of strings. Consecutive delimiters and delimiters at
> boundaries result in empty strings, unless strip\_empty is true.
> The default value of strip\_empty is false.
>
>
>
> **See also:** strtok.

— Function File: **strrep** (s, x, y)  
> Replace all occurrences of the substring x of the string s
> with the string y and return the result. For example,
>
> ```
>           strrep ("This is a test string", "is", "&%$")
>                => "Th&%$ &%$ a test string"
> ```
>
>
>
>
> **See also:** regexprep, strfind, findstr.

— Function File: **substr** (s, offset, len)  
> Return the substring of s which starts at character number
> offset and is len characters long.
>
> If offset is negative, extraction starts that far from the end of
> the string. If len is omitted, the substring extends to the end
> of S.
>
> For example,
>
> ```
>           substr ("This is a test string", 6, 9)
>                => "is a test"
> ```
>
> This function is patterned after AWK. You can get the same result by
> s`(`offset `: (`offset `+` len `- 1))`.

— Loadable Function: [s, e, te, m, t, nm] = **regexp** (str, pat)  
— Loadable Function: [...] = **regexp** (str, pat, opts, ...)  
> Regular expression string matching. Matches pat in str and
> returns the position and matching substrings or empty values if there are
> none.
>
> The matched pattern pat can include any of the standard regex
> operators, including:
>
> `.`: Match any character `* + ? {}`: Repetition operators, representing `*`: Match zero or more times `+`: Match one or more times `?`: Match zero or one times `{}`: Match range operator, which is of the form `{`n`}` to match exactly n times, `{`m`,}` to match m or more times, `{`m`,`n`}` to match between m and n times. `[...] [^...]`: List operators, where for example `[ab]c` matches `ac` and `bc` `()`: Grouping operator `|`: Alternation operator. Match one of a choice of regular expressions. The alternatives must be delimited by the grouping operator `()` above `^ $`: Anchoring operator. `^` matches the start of the string str and `$` the end
>
> In addition the following escaped characters have special meaning. It should
> be noted that it is recommended to quote pat in single quotes rather
> than double quotes, to avoid the escape sequences being interpreted by Octave
> before being passed to `regexp`.
>
> `\b`: Match a word boundary `\B`: Match within a word `\w`: Matches any word character `\W`: Matches any non word character `\<`: Matches the beginning of a word `\>`: Matches the end of a word `\s`: Matches any whitespace character `\S`: Matches any non whitespace character `\d`: Matches any digit `\D`: Matches any non-digit
>
> The outputs of `regexp` by default are in the order as given below
>
> s: The start indices of each of the matching substrings e: The end indices of each matching substring te: The extents of each of the matched token surrounded by `(...)` in pat. m: A cell array of the text of each match. t: A cell array of the text of each token matched. nm: A structure containing the text of each matched named token, with the name being used as the fieldname. A named token is denoted as `(?<name>...)`
>
> Particular output arguments or the order of the output arguments can be
> selected by additional opts arguments. These are strings and the
> correspondence between the output arguments and the optional argument
> are
>
> |  |  |  |  |
> | --- | --- | --- | --- |
> |  | 'start' | s |  |
> |  | 'end' | e |  |
> |  | 'tokenExtents' | te |  |
> |  | 'match' | m |  |
> |  | 'tokens' | t |  |
> |  | 'names' | nm |  |
>
> A further optional argument is 'once', that limits the number of returned
> matches to the first match. Additional arguments are
>
> matchcase: Make the matching case sensitive. ignorecase: Make the matching case insensitive. stringanchors: Match the anchor characters at the beginning and end of the string. lineanchors: Match the anchor characters at the beginning and end of the line. dotall: The character `.` matches the newline character. dotexceptnewline: The character `.` matches all but the newline character. freespacing: The pattern can include arbitrary whitespace and comments starting with `#`. literalspacing: The pattern is taken literally.
>
>
>
>
> **See also:** regexpi, regexprep.

— Loadable Function: [s, e, te, m, t, nm] = **regexpi** (str, pat)  
— Loadable Function: [...] = **regexpi** (str, pat, opts, ...)  
> Case insensitive regular expression string matching. Matches pat in
> str and returns the position and matching substrings or empty values
> if there are none. See regexp, for more details

— Loadable Function: string = **regexprep** (string, pat, repstr, options)  
> Replace matches of pat in string with repstr.
>
> The replacement can contain `$i`, which substitutes
> for the ith set of parentheses in the match string. E.g.,
>
> ```
>              regexprep("Bill Dunn",'(\w+) (\w+)','$2, $1')
> ```
>
> returns "Dunn, Bill"
>
> options may be zero or more of
>
> once: Replace only the first occurrence of pat in the result. warnings: This option is present for compatibility but is ignored. ignorecase or matchcase: Ignore case for the pattern matching (see `regexpi`). Alternatively, use (?i) or (?-i) in the pattern. lineanchors and stringanchors: Whether characters ^ and $ match the beginning and ending of lines. Alternatively, use (?m) or (?-m) in the pattern. dotexceptnewline and dotall: Whether . matches newlines in the string. Alternatively, use (?s) or (?-s) in the pattern. freespacing or literalspacing: Whether whitespace and # comments can be used to make the regular expression more readable. Alternatively, use (?x) or (?-x) in the pattern.
>
>
>
>
> **See also:** regexp, regexpi, strrep.

— Function File: **regexptranslate** (op, s)  
> Translate a string for use in a regular expression. This might
> include either wildcard replacement or special character escaping.
> The behavior can be controlled by the op that can have the
> values
>
> "wildcard": The wildcard characters `.`, `*` and `?` are replaced with wildcards that are appropriate for a regular expression. For example: ``` regexptranslate ("wildcard", "*.m") => ".*\.m" ``` "escape": The characters `$.?[]`, that have special meaning for regular expressions are escaped so that they are treated literally. For example: ``` regexptranslate ("escape", "12.5") => "12\.5" ```
>
>
>
>
> **See also:** regexp, regexpi, regexprep.
